# A Systematic Review and Meta-Analysis of Epidemiology and Clinical Manifestations of Human Brucellosis in China

**DOI:** 10.1155/2018/5712920

**Published:** 2018-04-22

**Authors:** Rongjiong Zheng, Songsong Xie, Xiaobo Lu, Lihua Sun, Yan Zhou, Yuexin Zhang, Kai Wang

**Affiliations:** ^1^Department of Infectious Diseases, The First Affiliated Hospital of Xinjiang Medical University, Urumqi, Xinjiang 830054, China; ^2^Department of Medical Engineering and Technology, Xinjiang Medical University, Urumqi, Xinjiang 830011, China

## Abstract

*Background*. Brucellosis has a wide spectrum of clinical manifestations and it may last several days or even several years; however, it is often misdiagnosed and therefore may cause inadequate therapy and prolonged illness. Previous studies about meta-analysis of manifestations of brucellosis reported in English lacked the data published in Chinese, which did not provide details about the contact history, laboratory tests, and misdiagnosis. We undertake a meta-analysis of clinical manifestations of human brucellosis in China to identify those gaps in the literature. We have searched published articles in electronic databases up to December 2016 identified as relating to clinical features of human brucellosis in China. 68 studies were included in the analysis. The main clinical manifestations were fever, fatigue, arthralgia, and muscle pain (87%, 63%, 62%, and 56%, resp.). There are significant differences between adults and children. Rash, respiratory and cardiac complications, and orchitis/epididymitis were more prevalent in children patients. The common complications of brucellosis were hepatitis, followed by osteoarthritis, respiratory diseases, cardiovascular diseases, central nervous system dysfunction, hemophagocytic syndrome, and orchitis/epididymitis in male. In the nonpastoral areas, brucellosis has a high ratio of misdiagnosis. Our analysis provides further evidence for the accurate diagnosis, particularly in assessing severe, debilitating sequelae of this infection.

## 1. Introduction

Brucellosis is one of the most common zoonotic infections globally [1, 2]. The disease is transmitted to humans by direct/indirect contact with infected animals or through the consumption of raw meat and dairy products [3, 4]. The main transmission routes are digestive tract, skin, and mucosal and respiratory tract contact with blood body fluids and aerosols.

Brucellosis has a wide spectrum of clinical manifestations, often lacks specificity, may last from several days to more than a year, is often misdiagnosed, and therefore causes inadequate therapy and prolonged illness can cause a severely debilitating and disabling illness. Patients may show fever, sweating, fatigue, and osteoarthritis [5] and even more serious conditions in different organ systems [6]. Brucellosis not only causes huge economic loss to the society by influencing the production of animal husbandry, but also threatens the human's physical and mental health [7].

Brucellosis was first reported in China in 1905 [8]. In recent years, human brucellosis incidence has increased sharply [9, 10]. Nationwide surveillance data indicated that the total incidence rate of human brucellosis in mainland China increased from 0.92 cases/100,000 people in 2004 to 4.2 cases/100,000 people in 2014 [11–13]. Currently, human brucellosis remains one of major public health issues in China.

This study presents a systematic review of scientific literature published before December 2016 identified as relating to clinical features of brucellosis in China. The objectives of this review were to identify those gaps in the literature of epidemiology, clinical manifestations, contact history, laboratory tests, and misdiagnosis of human brucellosis in China and provide further evidence for the accurate diagnosis, particularly in assessing severe, debilitating sequelae of human brucellosis.

## 2. Methods 

### 2.1. Search Strategy

We performed a systematic review of the literature to identify articles relating to clinical features of human brucellosis in China. With assistance of a professional medical librarian we electronically searched the literature in Wan Fang Data, Wei Pu Data, CNKI, Medline, Cochrane Library, and PubMed with MESH and keyword subject headings “brucellosis,” “malta fever,” “brucella melitensis,” or “brucella abortus,” AND “symptom,” “sequelae,” “morbidity,” “mortality,” “transmission mode,” “foodborne,” and “China,” for entries published from databases' inception before December 2016. We did not restrict the types of studies and publication languages. Duplicate entries were identified by two investigators screening the titles and abstracts of the article, the author, the year of publication, and the volume, issue, and page numbers of the source, and reviewing potentially relevant articles in full.

### 2.2. Selection Criteria

We systematically and inclusively reviewed articles by two investigators. The reviewers selected articles first by title and abstract, next by full text, and last by analyzing eligible studies in detail until demonstrating 100% agreement in articles included and excluded by two investigators.

Studies with the following criteria were excluded: (A) articles related to non-human brucellosis; (B) reported data that overlapped with already included articles; (C) articles that could not provide original data of the patients; (D) articles addressing topics that were not related to the clinical features of human brucellosis, such as treatment intervention and experimental laboratory studies.

Studies with the following criteria were included: (A) the literatures that described the clinical symptoms/syndromes of human brucellosis and the number of study subjects must more than 10 in each document; (B) the subjects reported in the literature who must be in China; (C) studies that provided data from general brucellosis cases and presented relevant laboratory results.

### 2.3. Data Extraction

Data was extracted by two reviewers independently including data collection, study design, study location, patient characteristics, the number of male and female patients, clinical manifestations, numbers of subjects with each symptom and complication which were recorded for each study, methods of diagnosis, and laboratory parameters. For the sex-related outcomes of epididymo/orchitis, the study population was considered to be only the male subgroups of the study population. Children patients must be of the age of 0–15 years. We also recorded the information relating to duration of illness prior to treatment, diagnostic delay, and exposure to potential risk factors. The results of data extraction must reach an agreement and consensus between the reviewers.

### 2.4. Statistical Analyses

We defined an event rate as the ratio of number of reported cases with a specific clinical manifestation to the total number of reported cases in each study. R statistical software (version 3.4.2, meta package) will be used for creating Forest plots to summarize composite data, generating proportions and corresponding 95% confidence intervals for each manifestation. Two-sided *P* values < 0.05 will be considered statistically significant during hypothesis testing.

## 3. Results

### 3.1. Systematic Review

**Figure 1:**
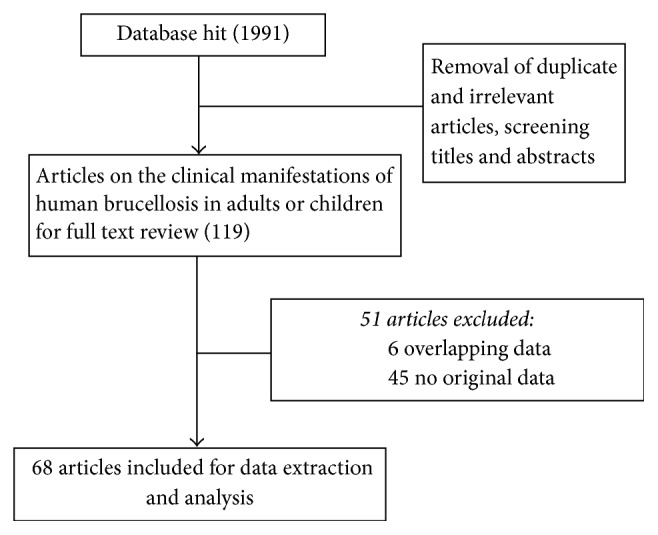
Procedure of the selection process.

Literature searches yielded 1991 potential articles, leaving 68 publications that met inclusion and exclusion criteria for data extraction and final analyses. 68 studies represented 12842 patients with human brucellosis in China. The male : female ratio was 2.64 : 1. All 68 articles included in the analysis were case series studies.  Figure 1 illustrates the detailed search process.

**Figure 2:**
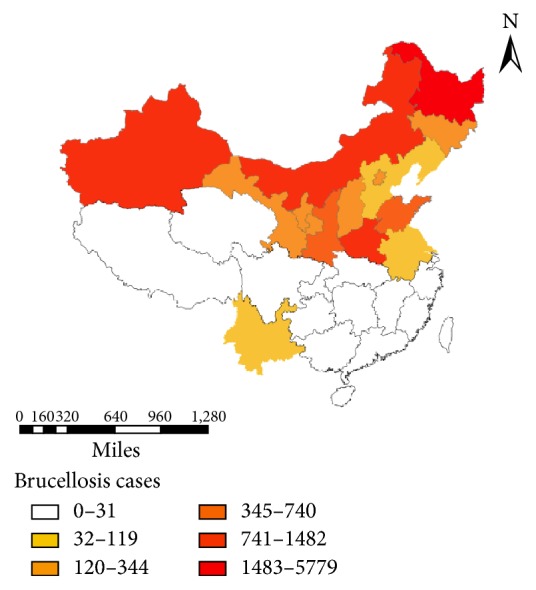
The geographic distribution of the numbers of subjects from each selected study.

Studies selected from 20 provinces or autonomous regions of China, including 39 studies from pastoral areas (12 from Xinjiang, 9 from Heilongjiang, 7 from Inner Mongolia, 4 from Jilin, 4 from Ningxia, 2 from Gansu, and 1 from Liaoning) and 29 studies from nonpastoral areas (6 from Shandong, 5 from Beijing, 3 from Henan, 3 from Shanxi, 2 from Hebei, 2 from Shaanxi, 2 from Tianjin, 1 from Guangdong, 1 from Hunan, 1 from Jiangsu, 1 from Jiangxi, 1 from Yunnan, and 1 from Zhejiang). The geographic distributions of the numbers of subjects from each selected study are shown in Figure 2.

We identified 41 studies which included both children and adult patients [14–54]. 10 studies investigated children with an upper age limit ranging from 0 months to 15 years [55–64]. 17 studies were about the adults who are more than 15 years old [65–81]. The results are presented in detail in Table 1.

### 3.2. Contact History

54 studies provided data about contact history (see Table 2). Most of the patients (79.4% [95% CI 76.5%–82.4%]) had histories of closely contacting with cattle, sheep, pigs, and dogs. 11.5% (95% CI 8.4%–15.7%) cases had consumption history of uncooked meat or dairy products. 16.7% (95% CI 13.5%–20.8%) cases got the infection of brucellosis with unknown reason. The brucellosis is mostly associated with direct/indirect contact with infected animals or through the consumption of animal products in China.

### 3.3. Clinical Syndromes and Complications

Table 3 shows the clinical syndromes and complications of patients by age category. There are 17 articles specifically describing the clinical characteristics of adult brucellosis representing 503 patients (male 408, female 95). Fever was the most common clinical syndrome (pooled rate 99% [95% CI 97%–100%]), followed by muscle pain (76% [95% CI 60%–95%]), fatigue (64% [95% CI 55%–74%]), arthralgia (61% [95% CI 52%–70%]), and sweating (57% [95% CI 48%–68%]). 10 articles specifically describe the clinical characteristics of children brucellosis including 384 patients (male 249, female 135). The most common symptoms of children patients were fever (92% [95% CI 87%–97%]), fatigue (68% [95% CI 56%–83%]), sweating (60% [95% CI 45%–79%]), and arthralgia (52% [95% CI 43%–64%]). The remaining 41 articles include pediatric and adult patients, a total of 11955 cases (male 8654, female 3301). Children patients have a higher incidence rate of rash, respiratory and cardiac complications, and orchitis/epididymitis. The morbidity of chills, headache, and weight loss are lower compared to adults.

Hepatitis (45% [95% CI 38%–54%]) and osteoarthritis (22% [95% CI 17%–29%]) were the most common complications. Central nervous system dysfunction (5% [95% CI 3%–10%]) which happened in overall patients included meningitis, encephalitis, cerebral infarction, and brain abscess. Cardiovascular diseases (9% [95% CI 6%–16%]) which were reported in overall patients involved the myocarditis, endocarditis, valvular neoplasm, valvular perforation, pericardial effusion, and heart failure. Hemophagocytic syndrome (6% [95% CI 2%–23%]) was only reported in adult patients. There were 13% of patients (95% CI: 7%–21%) suffering from respiratory manifestations, including cough, pneumonia, bronchial pneumonia, pleural effusion, respiratory failure, and pulmonary embolism. Orchitis or epididymitis occurred in 9% of the male patients (95% CI: 7%–12%).

### 3.4. Laboratory Tests

Table 4 shows the meta-analysis of the incidence of laboratory tests. There are 37 articles providing data of laboratory indicators of patients including 2999 cases. The mainly common reported abnormal laboratory tests were aleucocytosis (24.1% [95% CI 19.5%–29.8%]), anemia (23.9% [95% CI 18.5%–30.9%]), thrombocytopenia (15.8% [95% CI 12.7%–19.8%]), pancytopenia (13.2% [95% CI 9.3%–18.7%]), and leukocytosis (10.6% [95% CI 8.2%–13.7%]). The agglutination test was positive in 100% cases. Totally 30 articles including 4681 cases were tested by blood culture and* Brucella melitensis* species were isolated from (48.3% [95% CI 41.5%–56.3%]) cases.

### 3.5. Misdiagnosis

**Figure 3:**
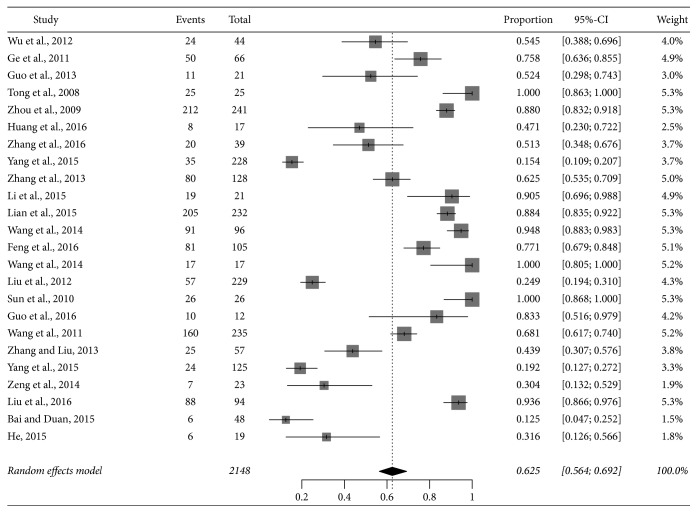
Forest plot of the incidence of misdiagnosis.

There are 24 articles that provided information of misdiagnosis of patients including 2148 cases. 10 studies were from pastoral areas (3 from Xinjiang, 2 from Heilongjiang, 2 from Ningxia, 1 from Inner Mongolia, and 2 from Jilin) and 14 studies from nonpastoral areas (4 from Shandong, 3 from Beijing, 1 from Henan, 1 from Hebei, 1 from Hunan, 1 from Shanxi, 1 from Tianjin, 1 from Jiangxi, and 1 from Jiangsu). A total of 1287 (62.5% [95% CI 56.4%–69.2%]) patients were misdiagnosed at the first visit (Figure 3). The misdiagnosed cases mainly occurred in nonpastoral provinces. Most patients were easily misdiagnosed as cold, rheumatic fever, rheumatoid arthritis, typhoid fever and paratyphoid fever, tuberculosis, malaria, septicemia, and lumbar disc herniation at the first visit in Department of Rheumatology, Hematology, Orthopedics, or Respiration.

## 4. Discussion

Brucellosis is one of the most widespread zoonoses worldwide [82, 83]. The number of brucellosis patients is increasing year by year in China. Shi et al. [84] analyzed the incidence and spatial-temporal distribution of human brucellosis from 1955 to 2014 in China, and the report showed that human brucellosis had reemerged since the mid-1990s and the affected areas had expanded from northern pastureland provinces to southern coastal and southwestern areas since 2004. In China brucellosis has been increasingly causing huge economic loss, and it has been a population health problem in recent years.

In humans, brucellosis involved multiorgans with a complicated and various clinical presentations ranging from nonspecific to severe symptoms [85], which makes brucellosis easily misdiagnosed as other diseases. If a chronic phase is developed for a lack of timely diagnosis and treatment, the disease can lead to a high rate of disability. Since the clinical summary of the relevant cases in China is published in Chinese, these cases are not included in the study using meta-analysis of* Brucella* abroad [86]. We analyzed the literature of clinical manifestations of human brucellosis in China.

From our analyzed data, it shows that 57% selected studies from pastoral areas and 43% from nonpastoral and coastal areas, consistent with previous epidemiological findings that the disease affected areas have expanded from northern pastureland provinces to southern coastal and southwestern areas over the past decades in China, but brucellosis is still mainly popular in pastoral areas [87]. In this study, there are 79.4% of patients who had close contact with sick animals and 11.5% of cases had consumption of uncooked meat or dairy products, indicating that contacting with infected animals and consuming unsterile animal products are the main transmission routes in China.

In the study, we found that the main clinical manifestations of human brucellosis are fever, fatigue, arthralgia, and muscle pain. The most common clinical syndromes of adult patients are fever, muscle pain, arthralgia, and sweating. Similar to our study, in a systematic review of the clinical manifestations of human brucellosis [86], the authors found that fever, arthralgia, myalgia, and back pain affected around half of the patients (78%, 65%, 47%, and 45%, resp.). There is controversy about whether clinical manifestations in children are significantly different from manifestations in adults. It had been reported in the literature that there was no significant difference in clinical manifestations between children and adults [88], which was very different in different literature. Some scholars reported that enlarged lymph nodes, spleen and liver, skin rashes, pharyngitis [89], and hematological and respiratory complications were more frequently observed in children than in adults [90]. Children had higher rates of hepatitis, osteoarticular manifestations [91], and lower rates of meningitis, endocarditis, spondylitis, and the progression to chronicity [92]. In the study, we found clinical differences between children and adults. Children had higher rates of rash, respiratory and cardiac complications, and orchitis/epididymitis. We also noted that chills, headache, and weight loss are less frequently observed in children patients.

Multiorgan involvement of* Brucella* is probably underrecognised [93]. Bone, CNS, and epididymis are the most commonly included organs [6]. The results of the current study were similar to those in other reported articles [94]. In the current study, results show that hepatitis and osteoarthritis were the more frequent complications. Serious complications such as central nervous system dysfunction, cardiovascular diseases, respiratory manifestations, and hemophagocytic syndromes are also observed. Orchitis or epididymitis occurred in 9% of the male patients. Brucellosis complications remain a major medical problem and it must still be regarded as a serious health problem in China.

In the study, results show that there is a high rate of misdiagnosis that mainly occurred in nonpastoral areas. Because of these manifestations such as fever, back pain, cough, gastrointestinal symptoms, and blood abnormalities, brucellosis is often misdiagnosed. Most misdiagnosed patients were admitted in Department of Rheumatology, Hematology, Orthopedics, and Respiration at the first visit.* Brucella* bacteria culture is the “gold standard” for the diagnosis of brucellosis [95, 96]. In the study, 87% of brucellosis patients have fever. However, we found that only 30 articles including 4681 cases were tested by blood culture and 48.3% of cases were positive of* Brucella melitensis*, indicating it may be the main reason of inappropriate diagnosis and inadequate therapy. Therefore, in order to reduce the rate of misdiagnosis effectively especially in the nonpastoral areas and provinces with high incidence of tuberculosis, it is necessary to broaden the ideas of clinical diagnosis with detailed history and carried out agglutination test and blood culture as early as possible for fever patients. One challenge in diagnosis of brucellosis is that the most common laboratory abnormalities are nonspecific. Most patients have normal blood cell counts on presentation. In the study, we found that the common abnormal laboratory tests were aleucocytosis (24.1%), anemia (23.9%), thrombocytopenia (15.8%), pancytopenia (13.2%), and leukocytosis (10.6%). In case of pancytopenia, the diagnosis of secondary hemophagocytosis should be considered. This condition may be triggered by* Brucella* and other intracellular pathogens [97, 98].

Our study has some limitations. First, although the incidence of brucellosis is very high in our country, the quantity and quality of articles reported in some provinces are not high, which leads to partial data omission. We failed to obtain more precise analysis of different clinical stages of brucellosis because part of the included literature did not clearly describe the brucellosis clinical stage and age classification. Second, most of the reported literatures lack detailed data on patient treatment options and prognosis, which results in the failure of analyzing therapeutic effect and prognosis.

In summary, we found that brucellosis was mainly popular in pastoral areas, but the disease affected areas had expanded from northern pastureland provinces to southern coastal and southwestern areas in China. The infection is mostly associated with the contact with infected animals or through the consumption of raw animal products. Clinical symptoms include fever, fatigue, arthralgia, sweating, and muscle pain with complication such as osteoarthritis, hepatitis, central nervous system dysfunction, cardiovascular diseases, respiratory manifestations, orchitis or epididymitis, and hemophagocytic syndromes. Further research is needed to characterize the analysis for therapeutic effect and prognosis of brucellosis in China. Our study provides initial evidence for the accurate diagnosis.

## Figures and Tables

**Table 1:** Main characteristics of all studies included in the meta-analysis.

First author & ref. number	Year	Age category	Location	Cases	Available contact history data	Available laboratory data	Available blood culture data	Available misdiagnosis data
Wu et al. [14]	2012	All ages	Beijing	44	Yes	NA	NA	Yes
Dai et al. [15]	2013	All ages	Beijing	23	Yes	Yes	Yes	NA
Ge et al. [16]	2011	All ages	Beijing	66	Yes	Yes	Yes	Yes
Tong et al. [17]	2013	All ages	Beijing	35	Yes	NA	NA	NA
Guo and Xu [18]	2013	All ages	Beijing	21	Yes	Yes	Yes	Yes
Wang et al. [19]	2015	All ages	Gansu	61	Yes	Yes	NA	NA
Gao et al. [20]	2002	All ages	Gansu	182	Yes	NA	NA	NA
Zhang et al. [21]	2012	All ages	Henan	21	Yes	Yes	Yes	NA
Li et al. [22]	2016	All ages	Henan	905	Yes	NA	NA	NA
Zhou [23]	2009	All ages	Henan	241	NA	NA	NA	Yes
Li et al. [24]	2008	All ages	Heilongjiang	165	Yes	Yes	Yes	NA
Liu and Zhang [25]	2016	All ages	Inner Mongolia	44	NA	NA	NA	NA
Liu et al. [26]	2015	All ages	Heilongjiang	314	NA	Yes	Yes	NA
Meng et al. [27]	2015	All ages	Heilongjiang	3318	NA	NA	Yes	NA
Gong et al. [28]	2010	All ages	Heilongjiang	1470	NA	NA	NA	NA
Liu et al. [29]	2012	All ages	Heilongjiang	229	Yes	Yes	Yes	Yes
Sun et al. [30]	2010	All ages	Jilin	270	Yes	NA	NA	NA
Wang et al. [31]	2014	All ages	Liaoning	88	Yes	NA	NA	NA
Xie et al. [32]	2016	All ages	Inner Mongolia	166	NA	NA	NA	NA
Sheng and Ma [33]	2009	All ages	Inner Mongolia	829	NA	NA	NA	NA
Sun et al. [34]	2014	All ages	Inner Mongolia	126	Yes	Yes	Yes	NA
W. Yang and F. Yang [35]	2015	All ages	Inner Mongolia	228	Yes	Yes	NA	Yes
Duan [36]	2015	All ages	Ningxia	57	Yes	NA	NA	NA
Zhang and Wang [37]	2013	All ages	Ningxia	128	Yes	NA	NA	Yes
Wang [38]	2005	All ages	Shandong	62	Yes	NA	NA	NA
Wang and Xiong [39]	2011	All ages	Shandong	235	NA	Yes	NA	Yes
Lian et al. [40]	2015	All ages	Shandong	232	Yes	NA	NA	Yes
Gao [41]	2016	All ages	Shandong	94	Yes	NA	NA	NA
Wang et al. [42]	2014	All ages	Shandong	96	Yes	NA	Yes	Yes
Wang [43]	2010	All ages	Shanxi	86	Yes	NA	NA	NA
Feng and Deng [44]	2016	All ages	Shanxi	105	Yes	Yes	Yes	Yes
An et al. [45]	2001	All ages	Shaanxi	622	Yes	NA	NA	NA
Zhang et al. [46]	2016	All ages	Yunnan	43	Yes	Yes	Yes	NA
Guo et al. [47]	2016	All ages	Xinjiang	124	Yes	NA	NA	NA
Pan et al. [48]	2013	All ages	Xinjiang	153	Yes	Yes	NA	NA
Zhang and Liu [49]	2013	All ages	Xinjiang	57	Yes	Yes	Yes	Yes
Yang et al. [50]	2015	All ages	Xinjiang	125	NA	NA	NA	Yes
Zhang [51]	2016	All ages	Xinjiang	191	Yes	Yes	NA	NA
Ju et al. [52]	2011	All ages	Xinjiang	156	Yes	Yes	Yes	NA
Gao et al. [53]	2012	All ages	Xinjiang	426	NA	NA	NA	NA
Wang et al. [54]	2015	All ages	Xinjiang	117	Yes	Yes	Yes	NA
Wang et al. [55]	2014	Children	Hebei	80	Yes	NA	NA	NA
Zeng et al. [56]	2014	Children	Jilin	23	Yes	Yes	Yes	Yes
Wang et al. [57]	2016	Children	Xinjiang	16	Yes	Yes	NA	NA
Fan et al. [58]	2016	Children	Xinjiang	24	Yes	Yes	NA	NA
Zhang et al. [59]	2006	Children	Jilin	25	NA	Yes	NA	NA
Yu et al. [60]	2012	Children	Heilongjiang	38	NA	Yes	NA	NA
Lu and Liu [61]	2015	Children	Inner Mongolia	17	Yes	Yes	NA	NA
Liu et al. [62]	2016	Children	Heilongjiang	94	Yes	Yes	Yes	Yes
Bai and Duan [63]	2015	Children	Ningxia	48	Yes	Yes	Yes	Yes
He [64]	2015	Children	Xinjiang	19	Yes	Yes	Yes	Yes
Zheng et al. [65]	2016	Adults	Guangdong	12	Yes	Yes	Yes	NA
Tong et al. [66]	2008	Adults	Hebei	25	Yes	Yes	Yes	Yes
Chen and Dong [67]	2016	Adults	Heilongjiang	60	Yes	NA	NA	NA
Huang [68]	2016	Adults	Hunan	17	Yes	Yes	Yes	Yes
Ji et al. [69]	2006	Adults	Heilongjiang	30	NA	Yes	Yes	NA
M. Wang and L. Wang [70]	2007	Adults	Jilin	26	Yes	Yes	NA	Yes
Zhang et al. [71]	2016	Adults	Jiangsu	39	Yes	Yes	Yes	Yes
Guo et al. [72]	2016	Adults	Jiangxi	12	Yes	Yes	Yes	Yes
Zhang [73]	2011	Adults	Inner Mongolia	27	Yes	Yes	NA	NA
Yan et al. [74]	2016	Adults	Ningxia	31	NA	Yes	Yes	NA
Li et al. [75]	2015	Adults	Shandong	21	Yes	Yes	Yes	Yes
Wu et al. [76]	2007	Adults	Shanxi	28	Yes	Yes	NA	NA
Zhang and Li [77]	2015	Adults	Shaanxi	35	Yes	Yes	Yes	NA
Wang [78]	2014	Adults	Tianjin	17	Yes	Yes	Yes	Yes
Zhou and Yang [79]	2014	Adults	Tianjin	18	Yes	Yes	Yes	NA
Xu et al. [80]	2007	Adults	Zhejiang	31	Yes	NA	NA	NA
Chen et al. [81]	2016	Adults	Xinjiang	74	Yes	Yes	NA	NA

**Table 2:** Meta-analysis of the contact history.

Contact	*n*	Proportion [95% CI]
Contact history	54	0.794 [0.7651; 0.8240]
Digestive tract contact	31	0.115 [0.0844; 0.1567]
Unknown	43	0.167 [0.1347; 0.2077]

**Table 3:** Meta-analysis of clinical manifestations of brucellosis by age category.

Manifestation	Age category						All studies	
	Children		Adults		All ages			
General	*n*	% [95% CI]	*n*	% [95% CI]	*n*	% [95% CI]	*n*	% [95% CI]
Fever	10	92 [87; 97]	17	99 [97; 100]	41	83 [80; 87]	68	87 [85; 90]
Fatigue	7	68 [56; 83]	14	64 [55; 74]	34	62 [57; 67]	55	63 [59; 67]
Chills	3	26 [8; 82]	5	53 [36; 79]	4	37 [33; 42]	12	43 [33; 55]
Sweats	8	60 [45; 79]	16	57 [48; 68]	39	54 [49; 59]	63	55 [51; 60]
Arthralgia	9	52 [43; 64]	17	61 [52; 70]	40	63 [59; 68]	66	62 [58; 65]
Headache	4	8 [3; 19]	10	29 [19; 42]	27	21 [18; 25]	41	21 [18; 25]
Muscle pain	2	31 [7; 100]	5	76 [60; 95]	20	53 [47; 59]	27	56 [51; 62]
Nausea/vomiting	6	27 [16; 43]	8	26 [15; 45]	17	25 [19; 34]	31	26 [21; 33]
Rash	3	13 [6; 29]	3	7 [3; 19]	9	5 [3; 11]	15	7 [4; 11]
Weight loss	0	-	4	26 [14; 47]	5	32 [17; 61]	9	29 [17; 48]
Skin petechia	3	8 [4; 18]	2	18 [10; 32]	9	5 [3; 8]	14	7 [4; 10]
Abdominal pain	2	6 [1; 31]	3	6 [3; 14]	3	8 [4; 16]	8	8 [5; 11]
Chest pain	0	-	2	7 [3; 17]	1	5 [3; 10]	3	6 [3; 10]
Cough	5	12 [8; 17]	4	19 [12; 29]	5	10 [8; 14]	14	12 [10; 15]
Hepatomegaly	7	28 [18; 42]	7	23 [13; 40]	23	13 [10; 17]	37	16 [13; 20]
Splenomegaly	7	35 [27; 45]	10	29 [22; 39]	23	21 [16; 27]	40	24 [20; 29]
Lymphadenectasis	7	38 [25; 58]	7	32 [22; 48]	27	16 [12; 21]	41	19 [15; 25]
Hepatitis	8	48 [34; 67]	15	60 [52; 69]	24	38 [30; 49]	47	45 [38; 54]
Neurological	4	8 [4; 17]	3	8 [2; 36]	14	4 [2; 9]	21	5 [3; 10]
Cardiac	3	19 [2; 100]	2	5 [1; 19]	12	9 [6; 14]	17	9 [6; 16]
Hemophagocytic syndrome	0	-	0	-	4	6 [2; 23]	4	6 [2; 23]
Respiratory	5	26 [12; 57]	3	11 [6; 20]	8	9 [4; 23]	16	13 [7; 21]
Orchitis/ epididymitis	1	67 [45; 100]	7	6 [3; 12]	34	9 [7; 12]	42	9 [7; 12]
Osteoarthritis	2	16 [8; 35]	4	22 [9; 52]	11	23 [17; 31]	17	22 [17; 29]

**Table 4:** Meta-analysis of the incidence of laboratory tests.

Laboratory	The number of articles	Proportion [95% CI]
Thrombocytopenia	32	0.158 [0.1268; 0.1979]
Aleucocytosis	37	0.241 [0.1951; 0.2984]
Leukocytosis	16	0.106 [0.0819; 0.1365]
Anemia	28	0.239 [0.1847; 0.3094]
Pancytopenia	6	0.132 [0.093; 0.187]
